# The LISA-Taiji Network: Precision Localization of Coalescing Massive Black Hole Binaries

**DOI:** 10.34133/2021/6014164

**Published:** 2021-01-06

**Authors:** Wen-Hong Ruan, Chang Liu, Zong-Kuan Guo, Yue-Liang Wu, Rong-Gen Cai

**Affiliations:** ^1^CAS Key Laboratory of Theoretical Physics, Institute of Theoretical Physics, Chinese Academy of Sciences, P.O. Box 2735, Beijing 100190, China; ^2^School of Physical Sciences, University of Chinese Academy of Sciences, No. 19A Yuquan Road, Beijing 100049, China; ^3^School of Fundamental Physics and Mathematical Sciences, Hangzhou Institute for Advanced Study, University of Chinese Academy of Sciences, Hangzhou 310024, China

## Abstract

We explore a potential LISA-Taiji network to fast and accurately localize the coalescing massive black hole binaries. For an equal-mass binary located at redshift of 1 with a total intrinsic mass of 10^5^*M*_⊙_, the LISA-Taiji network may achieve almost four orders of magnitude improvement on the source localization region compared to an individual detector. The precision measurement of sky location from the gravitational-wave signal may completely identify the host galaxy with low redshifts prior to the final black hole merger. Such identification of the host galaxy is vital for the follow-up variability in electromagnetic emissions of the circumbinary disc when the binary merges to a new black hole and enables the coalescing massive black hole binaries to be used as a standard siren to probe the expansion history of the Universe.

## 1. Introduction

**Figure 1:**
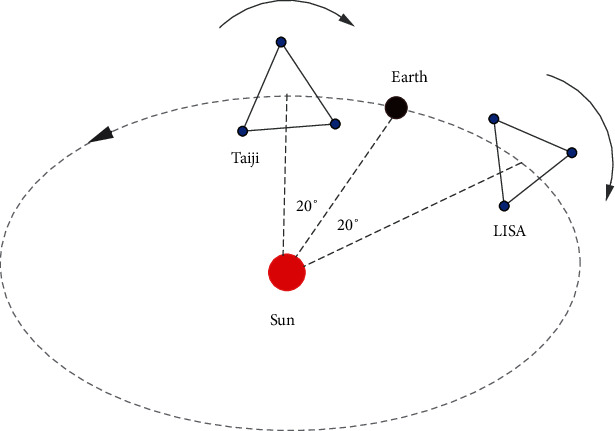
Configuration of the LISA-Taiji network. The LISA constellation moves in a heliocentric orbit behind the Earth by about 20° while the Taiji constellation orbits the Sun ahead of the Earth by about 20°. As expected, the LISA-Taiji network with a separation distance of about 0.7 AU can improve the sky localization of GW sources.

The Laser Interferometer Space Antenna (LISA), a collaborative ESA-NASA project, is proposed to detect gravitational waves (GWs) in a frequency range of 10^−4^ Hz to 10^−1^ Hz. LISA consists of a triangle of three spacecraft with a separation distance of 2.5 million kilometers in a heliocentric orbit. The constellation follows the Earth by about 20° (Figure 1). It is expected to launch during 2030-2035, with a mission lifetime of 4 years extendable to 10 years [1]. Like LISA, Taiji proposed by the Chinese Academy of Sciences is composed of a triangle of three spacecraft with a separation distance of 3 million kilometers in a heliocentric orbit ahead of the Earth by about 20° (Figure 1). Compared to LISA, Taiji is slightly more sensitive to low-frequency GWs due to its longer arms [2]. Since Taiji would launch in the same period [3], it is possible to combine these two space-based GW observatories into a LISA-Taiji network [4], which can improve the sky localization of GW sources due to the faraway separation of the two constellations (Figure 1). In this letter, we investigate for the first time the network's potential ability to localize GW sources (Notice that the localization capacity of the LISA-Taiji network was further studied in [5] after the present work appeared on arXiv.).

Fast and accurately localizing GW sources is one of the key tasks for both ground-based and space-based GW observations, which allow us to search for the follow-up electromagnetic counterparts and to uniquely identify the host galaxy. Once the host galaxy is identified from GW observations, one can easily read off the redshift of the source with a good accuracy from electromagnetic measurements. Measuring such GWs as potentially powerful standard sirens [6–8] provides detailed information on the high-redshift expansion history of the Universe and offers an independent way of measuring cosmological parameters.

For a single ground-based GW detector, it is hard to localize the sky position of a transient GW signal from a stellar-mass black hole binary because the detector is sensitive to GWs from nearly all directions. Two joint detectors can in principle restrict the position of the source to an annulus in the sky by triangulation using the time difference on arrival at the two detectors. However, with a network of more than two detectors, the sky position of the source can be inferred by triangulation, phase differences, and amplitude ratios on arrival at the detectors. Wen and Chen derive geometrical expressions for the angular resolution of a network of GW detectors [9]. Recently, the addition of the Advanced Virgo detector to the LIGO detector network significantly improves the sky localization of GW170814, reducing the 90% credible area on the sky from 1160 deg^2^ using only the two Advanced LIGO detectors to 60 deg^2^ using the LIGO-Virgo network [10].

For space-based GW observatories such as LISA and Taiji, a single detector is able to localize the sky position of GW sources including massive black hole binaries (MBHBs), extreme mass ratio inspirals, and compact binaries in the Milky Way, by the motion of the detector in space. Actually, the detector can be effectively regarded as a network including a set of detectors at different locations along the detector's trajectory in space, which observe a given GW event at different times. In general, such GW signals are observed by the detector for several days, months, or even years. For instance, the coalescence of a MBHB with a total mass of 10^5^*M*_⊙_ lasts for several months in the frequency band of the detector. The time dependence of the antenna pattern functions plays a crucial role in localizing these GW sources [11]. Similar to the LIGO-Virgo network, the LISA-Taiji network is expected to improve localization due to their separation distance of about 0.7 AU.

## 2. Results

In our analysis, we focus on coalescing MBHBs with total masses between 10^4^*M*_⊙_ and 10^8^*M*_⊙_, which are the strongest GW sources for space-based GW observatories. There are some pieces of indirect evidence for the existence of MBHBs in galactic centers. Although the origin of massive black holes remains unclear, MBHBs inevitably form due to frequent galaxy mergers [12]. MBHBs with kpc scale separations have been unambiguously detected in optical and X-ray surveys [13]. However, observations of MBHBs with sub-pc scale separations are particularly challenging because these small separations at cosmic distance are well below the angular resolving power of the current telescopes. So far, only MBHB candidates have been found through optical variability with the periods of ~14 years in the center of Ark 120 [14] and ~20 years in the center of NGC 5548 [15]. Fortunately, when the orbital period of the system becomes smaller than hours, there is a good chance to detect MBHBs in galactic centers by GW measurements. Such GWs are expected to be observed by space-based GW observatories with a high signal-to-noise ratio (SNR).

Using the Fisher information matrix approach (see Methods), we analyze the sky localization for coalescing MBHBs with the LISA-Taiji network. In our analysis, we restrict our attention to the inspiral phase. Since the source position is localized mainly by triangulation using the time difference on arrival at the two detectors, the sky localization critically relies on long integration times. The sky localization from inspiral could be used to search for the follow-up electromagnetic variability associated with the final merger. The inspiral GW signal is modeled by a restricted post-Newtonian waveform (see Methods). The upper cutoff frequency is dictated by the innermost stable circular orbit. We consider an equal-mass black hole binary with a total intrinsic mass of 10^5^*M*_⊙_, located at redshift of *z* = 1.

**Figure 2:**
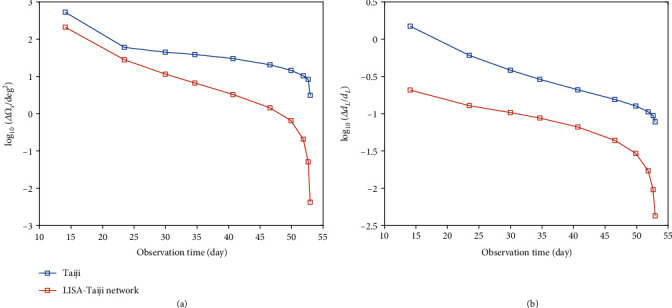
Measurements of the (a) angular resolution, Δ*Ω*_*s*_, and the (b) luminosity distance uncertainty, Δ*d*_*L*_/*d*_*L*_, from increasing length of observation time with Taiji (blue) and the LISA-Taiji network (red). We choose an equal-mass black hole binary, located at redshift of *z* = 1 with a total intrinsic mass of 10^5^*M*_⊙_.

In Figure 2, we show measurements of the angular resolution (Figure 2(a)), Δ*Ω*_*s*_, and the luminosity distance uncertainty (Figure 2(b)), Δ*d*_*L*_/*d*_*L*_, from increasing length of observation time with Taiji (blue) and the LISA-Taiji network (red). The GW signal can be observed about 52 days prior to merger, which is determined by its frequency segment falling in the detector band, with a SNR of 719 for Taiji and 943 for the LISA-Taiji network. From Figure 2, we can see that the measurement errors in the solid angle and luminosity distance decrease rapidly during the last days prior to the final merger. Significant improvement of the sky localization by the LISA-Taiji network over a single detector mainly comes from the short-duration inspiral near the merger instead of the long-duration inspiral far before the merger. Because the frequency of the GW signal changes rapidly in a few days before the merger, the signal contains much information, which can break the degeneracy between the sky location and other parameters. With Taiji, the source can be localized with Δ*Ω*_*s*_ < 4 deg^2^ and Δ*d*_*L*_/*d*_*L*_ < 8%, while with the LISA-Taiji network the source can be localized with Δ*Ω*_*s*_ < 0.005 deg^2^ and Δ*d*_*L*_/*d*_*L*_ < 0.5%. This indicates that the constraints on the solid angle are improved by three orders of magnitude and on the luminosity distance are improved by one order of magnitude. Therefore, the LISA-Taiji network may achieve almost four orders of magnitude improvement on the source localization region compared to an individual detector. Our further calculation indicates that the conclusion applies to the cases of 10^6^*M*_⊙_ and 10^7^*M*_⊙_.

**Figure 3:**
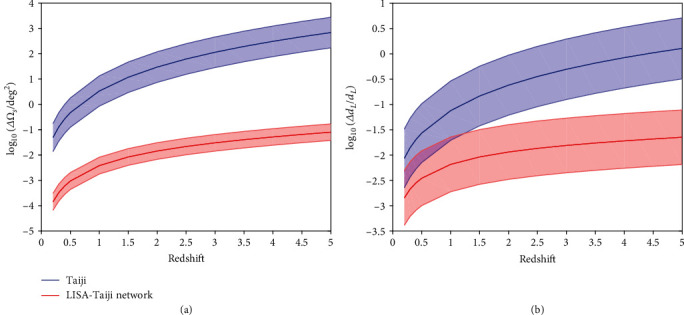
Measurements of the (a) angular resolution and the (b) luminosity distance uncertainty, as a function of redshifts of the equal-mass black hole binaries with a total intrinsic mass of 10^5^*M*_⊙_, with Taiji (blue) and the LISA-Taiji network (red). The center line denotes the median value while the shaded region denotes the 1*σ* uncertainties using a catalogue of 10,000 simulated sources with a fixed redshift at different sky positions.

With LISA or Taiji, the angular resolution is not good enough to identify the host galaxy from inspiral GW observations when spin-induced precession of MBHBs is negligible [1, 2, 11]. The LISA-Taiji network is able to completely identify the host galaxy with low redshifts prior to the final merger. In Figure 3, we show measurements of the angular resolution (Figure 3(a)) and the luminosity distance uncertainty (Figure 3(b)), as a function of redshifts of the equal-mass black hole binaries with a total intrinsic mass of 10^5^*M*_⊙_, with Taiji (blue) and the LISA-Taiji network (red). SNRs of 10,000 simulated sources at redshift of *z* = 1 range from 60 to 2600 for Taiji and from 270 to 3500 for the LISA-Taiji network. Assuming that galaxies are uniformly distributed in comoving volume with a number density of 0.02 Mpc^−3^, we estimate the number of potential galaxies within the source localization volume. We find that the LISA-Taiji network can identify the host galaxy of the MBHB with a total intrinsic mass of 10^5^*M*_⊙_ if the galaxy redshift is smaller than 0.75. Moreover, we consider more massive binaries. In the cases of 10^6^*M*_⊙_ and 10^7^*M*_⊙_, our calculation indicates that the host galaxies with *z* < 0.96 and *z* < 0.45 can be identified, respectively.

Actually, the measurements of the sky localization depend not only on the mass and redshift but also on the inclination angle and sky position of GW sources [16]. To investigate the effects of the inclination and sky position on the angular resolution, we simulate 10,000 random MBHB sources with a total intrinsic mass of 10^5^*M*_⊙_ at a fixed redshift (see Methods). The 1*σ* uncertainties induced by random source positions are shown in Figure 3. These uncertainties are modestly insensitive to the distance to sources.

We have investigated the ability of the LISA-Taiji network to localize the GW sources of MBHBs using the Fisher information matrix method. We find that the LISA-Taiji network achieves a remarkable ability improvement on the sky localization compared to an individual detector. It is possible to completely identify host galaxies only from GW detections prior to the final black hole merger. This provides us a good chance to measure possible variability of electromagnetic emissions of the circumbinary disc when the MBHB merges to a new black hole and allows us to explore the expansion of the Universe using MBHBs as standard sirens even without consequently electromagnetic counterparts.

The newly formed black hole suffers a recoil because GWs carry away a nonzero linear momentum. The effect of such a recoil on the circumbinary disc in principle gives rise to an electromagnetic counterpart to the merger [17]. However, predicting the spectrum and light curve remains to be a challenge. Whether the electromagnetic counterpart is detectable remains uncertain. In this sense, the precision localization from GW observations would play a crucial role in searching for the electromagnetic counterpart.

## 3. Methods

### 3.1. GW Waveforms and Detector Response Functions

The matched filter is used to search for the GW signal from data and to estimate the parameters of the GW source, which requires the waveform template of coalescing compact binaries. The GW signal from an inspiraling nonspinning MBHB can be modeled by a restricted post-Newtonian (PN) waveform (i.e., the amplitude is kept at the dominant Newtonian level while the phase is evolved to the second PN order). The effect of spin-induced precession on the sky localization of MBHBs is studied in [11]. Since we focus on improvement of the sky localization by the LISA-Taiji network over a single detector, the effect of spin-induced precession is ignored in our analysis. Two polarization amplitudes of the GW signal are given by

(1) $$\begin{array}{c} h_{+,\times }\left(t\right)=\frac{2GM_{c}\eta^{2/5}x}{c^{2}d_{L}}\left[H_{+,\times }^{\left(0\right)}+x^{1/2}H_{+,\times }^{\left(1/2\right)}+xH_{+,\times }^{\left(1\right)}+x^{3/2}H_{+,\times }^{\left(3/2\right)}+x^{2}H_{+,\times }^{\left(2\right)}+x^{5/2}H_{+,\times }^{\left(5/2\right)}+O\left(\frac{1}{c^{6}}\right)\right], \end{array}$$

where *d*_*L*_ is the luminosity distance to the source, *M*_*c*_ = *η*^3/5^*M* is the chirp mass, *M* = *m*_1_ + *m*_2_ is the total mass, and *η* = *m*_1_*m*_2_/*M*^2^ is the symmetric mass ratio. The invariant PN velocity parameter *x* is defined by

(2) $$\begin{array}{c} x\equiv {\left(\frac{GM\omega }{c^{3}}\right)}^{2/3}, \end{array}$$

where *ω* is the orbital frequency of the binary for a circular orbit. To the lowest PN order in the amplitude evolution, the waveform for *t* < *t*_*c*_ is given by

(3) $$\begin{array}{c} h_{+}\left(t\right)=-\frac{1+\cos^{2}\iota }{2}\left(\frac{GM_{c}}{c^{2}d_{L}}\right){\left(\frac{t_{c}-t}{5GM_{c}/c^{3}}\right)}^{-1/4}\cos\left[2\phi_{c}+2\phi \left(t-t_{c};M_{c},\eta \right)\right], \end{array}$$

(4) $$\begin{array}{c} h_{\times }\left(t\right)=-\cos\iota \left(\frac{GM_{c}}{c^{2}d_{L}}\right){\left(\frac{t_{c}-t}{5GM_{c}/c^{3}}\right)}^{-1/4}\sin\left[2\phi_{c}+2\phi \left(t-t_{c};M_{c},\eta \right)\right], \end{array}$$

where *ι* is the angle between the orbital angular momentum axis of the binary and the direction to the detector and *t*_*c*_ and *ϕ*_*c*_ are the coalescence time and coalescence phase. In the LISA-Taiji network, we choose the polar coordinate system with the Sun as its origin. So the strain on a detector is given by

(5) $$\begin{array}{c} h\left(t+\tau \right)=F_{+}\left(\theta ,\phi ,\psi \right)h_{+}\left(t\right)+F_{\times }\left(\theta ,\phi ,\psi \right)h_{\times }\left(t\right), \end{array}$$

where *F*_+_ and *F*_×_ are the detector response functions, *θ* and *ϕ* are the colatitude and longitude of the binary in the polar coordinate system (assuming that the center of mass of the binary is at rest), and *ψ* is the polarization angle. Here, *τ* is the delay between the arrival time of GWs at the Sun and the arrival time at the detector, which is given by

(6) $$\begin{array}{c} \tau =\frac{\vec{x}\left(t\right)\cdot \hat{k}}{c}, \end{array}$$

where $\vec{x}\left(t\right)$ is the position vector of the source relative to the detector and $\hat{k}$ is the unit vector from the source to the Sun. Therefore, the strain can be written as

(7) $$\begin{array}{c} h\left(t\right)=-\frac{GM_{c}}{c^{2}D_{\text{eff}}}{\left(\frac{t_{0}-t}{5GM_{c}/c^{3}}\right)}^{-1/4}\cos\left[2\phi_{0}+2\phi \left(t-t_{0};M_{c},\eta \right)\right], \end{array}$$

where $t_{0}=t_{c}+\tau \left(\vec{x}\left(t\right)\right)$ is the coalescence time at the detector, *ϕ*_0_ is

(8) $$\begin{array}{c} 2\phi_{0}=2\phi_{c}-\arctan\left(\frac{F_{\times }\left(\theta ,\phi ,\psi \right)}{F_{+}\left(\theta ,\phi ,\psi \right)}\frac{2\cos\iota }{1+\cos^{2}\iota }\right), \end{array}$$

and the effective luminosity distance to the source, *D*_eff_, is given by

(9) $$\begin{array}{c} D_{\text{eff}}=d_{L}{\left[F_{+}^{2}{\left(\frac{1+\cos^{2}\iota }{2}\right)}^{2}+F_{\times }^{2}\cos^{2}\iota \right]}^{-1/2}. \end{array}$$

The Fourier transformation of the strain (7) can be obtained using the stationary phase approximation. For a ground-based GW detector, *F*_+_, *F*_×_, and *τ* in (7) can be regarded as constants for a GW burst. In this case, the frequency-domain version of the strain reads

(10) $$\begin{array}{c} \tilde{h}\left(f\right)=-{\left(\frac{5\pi }{24}\right)}^{1/2}\left(\frac{GM_{c}}{c^{3}}\right)\left(\frac{GM_{c}}{c^{2}D_{\text{eff}}}\right){\left(\frac{GM_{c}}{c^{3}}\pi f\right)}^{-7/6}e^{-i\Psi \left(f;M_{c},\eta \right)}, \end{array}$$

where Ψ is written to the second PN order by

(11) $$\begin{array}{c} \Psi \left(f;M_{c},\eta \right)=2\pi ft_{0}-2\phi_{0}-\frac{\pi }{4}+\frac{3}{128\eta }\left[\nu^{-5}+\left(\frac{3715}{756}+\frac{55}{9}\eta \right)\nu^{-3}-16\pi \nu^{-2}+\left(\frac{15293365}{508032}+\frac{27145}{504}\eta +\frac{3085}{72}\eta^{2}\right)\nu^{-1}\right], \end{array}$$

(12) $$\begin{array}{c} \nu ={\left(\frac{G\pi M}{c^{3}}f\right)}^{1/3}. \end{array}$$

For space-based GW detectors such as LISA and Taiji, the observation time for a GW signal usually lasts for several days, months, or even years. Thus, the location change of the source to the detector cannot be ignored. In general, *F*_+_(*t*), *F*_×_(*t*), and $\tau \left(\vec{x}\left(t\right)\right)$ in (7) are functions of observation time. According to the forward modeling of LISA described in Ref. [18], to linear order in eccentricity, the time delay is given by

(13) $$\begin{array}{c} \tau \left(t\right)=-\frac{R}{c}\sin\theta \;\cos\left(\alpha -\phi \right)-\frac{eR}{2c}\sin\theta \left[\cos\left(2\alpha -\phi -\beta \right)-3\cos\left(\phi -\beta \right)\right], \end{array}$$

where *R* = 1 AU, *e* is the eccentricity of the detector's orbit, *β* = 2*πn*/3 (*n* = 0, 1, 2) is the relative phase of three spacecraft, and *α* = 2*πf*_*m*_*t* + *κ* is the orbital phase of the guiding center. Like LISA, Taiji is viewed as a combination of two independent detectors in our analysis. Here, *κ* is the initial ecliptic longitude of the guiding center and *f*_*m*_ = 1/yr. The detector response functions can be written as

(14) $$\begin{array}{c} F_{+}\left(t\right)=\frac{1}{2}\left(\cos\left(2\psi \right)D_{+}\left(t\right)-\sin\left(2\psi \right)D_{\times }\left(t\right)\right), \end{array}$$

(15) $$\begin{array}{c} F_{\times }\left(t\right)=\frac{1}{2}\left(\sin\left(2\psi \right)D_{+}\left(t\right)+\cos\left(2\psi \right)D_{\times }\left(t\right)\right). \end{array}$$

Using the low-frequency approximation, one has

(16) $$\begin{array}{c} D_{+}\left(t\right)=\frac{\sqrt{3}}{64}\left[-36\sin^{2}\theta \;\sin\left(2\alpha \left(t\right)-2\beta \right)+\left(3+\cos\left(2\theta \right)\right)\left(\cos\left(2\phi \right)\left(9\sin\left(2\beta \right)-\sin\left(4\alpha \left(t\right)-2\beta \right)\right)+\sin\left(2\phi \right)\left(\cos\left(4\alpha \left(t\right)-2\beta \right)-9\cos\left(2\beta \right)\right)\right)-4\sqrt{3}\sin\left(2\theta \right)\left(\sin\left(3\alpha \left(t\right)-2\beta -\phi \right)-3\sin\left(\alpha \left(t\right)-2\beta +\phi \right)\right)\right], \end{array}$$

(17) $$\begin{array}{c} D_{\times }\left(t\right)=\frac{1}{16}\left[\sqrt{3}\cos\theta \left(9\cos\left(2\phi -2\beta \right)-\cos\left(4\alpha \left(t\right)-2\beta -2\phi \right)\right)-6\sin\theta \left(\cos\left(3\alpha \left(t\right)-2\beta -\phi \right)+3\cos\left(\alpha \left(t\right)-2\beta +\phi \right)\right)\right]. \end{array}$$

The stationary phase approximation is employed to obtain the frequency-domain version of the strain given by the same as (10), in which *F*_+_, *F*_×_, and *τ* are replaced by [19]

(18) $$\begin{array}{c} F_{+}\left(f\right)=F_{+}\left(t=t_{f}\right),\\ F_{\times }\left(f\right)=F_{\times }\left(t=t_{f}\right),\\ \tau \left(f\right)=\tau \left(t=t_{f}\right), \end{array}$$

where

(19) $$\begin{array}{c} t_{f}=t_{c}-\frac{5GM}{256\eta c^{3}}\left[\nu^{-8}+\left(\frac{743}{252}+\frac{11}{3}\eta \right)\nu^{-6}-\frac{32\pi }{5}\nu^{-5}+\left(\frac{3058673}{508032}+\frac{5429}{504}\eta +\frac{617}{72}\eta^{2}\right)\nu^{-4}\right]. \end{array}$$

In our analysis, we only consider the leading term in equation (19). Like LISA, Taiji consists of a triangle of three identical spacecraft in the heliocentric orbit. Therefore, these results are applicable to Taiji.

### 3.2. Fisher Information Matrix Approach

If the strain is well modeled by the formulas obtained above, the parameter estimation from maximum likelihood test is close to the true value of the parameters and the errors can be estimated by the Fisher information matrix. For a network including *N* independent detectors, the Fisher information matrix can be written as

(20) $$\begin{array}{c} \Gamma_{ij}=\left(\frac{\partial_{i}d\left(f\right)}{\partial \lambda_{i}},\frac{\partial_{j}d\left(f\right)}{\partial \lambda_{j}}\right), \end{array}$$

where **d** is given by

(21) $$\begin{array}{c} d\left(f\right)={\left[\frac{{\tilde{h}}_{1}\left(f\right)}{\sqrt{S_{1}\left(f\right)}},\frac{{\tilde{h}}_{2}\left(f\right)}{\sqrt{S_{2}\left(f\right)}},\cdots ,\frac{{\tilde{h}}_{N}\left(f\right)}{\sqrt{S_{N}\left(f\right)}}\right]}^{\text{T}}, \end{array}$$

and *λ*_*i*_ denotes the parameters (*M*_*c*_, *η*, *d*_*L*_, *θ*, *ϕ*, *ι*, *t*_*c*_, *ϕ*_*c*_, *ψ*) for nonspinning MBHBs. Here, *S*_*i*_(*f*) is the noise power spectral density of the *i*th detector and ${\tilde{h}}_{i}\left(f\right)$ is the strain on it. The bracket in (20) for two functions *a*(*t*) and *b*(*t*) is defined as

(22) $$\begin{array}{c} \left(a,b\right)=2\int_{f_{\text{low}}}^{f_{\text{up}}}df\left[\tilde{a}\left(f\right){\tilde{b}}^{*}\left(f\right)+{\tilde{a}}^{*}\left(f\right)\tilde{b}\left(f\right)\right]. \end{array}$$

In our analysis, we choose *f*_up_ as the innermost stable circular orbit frequency, *f*_isco_, which is given by

(23) $$\begin{array}{c} f_{\text{isco}}=\frac{c^{3}}{6\sqrt{6}\pi GM}. \end{array}$$

The root-mean-squared (1*σ*) errors can be estimated by the Fisher information matrix

(24) $$\begin{array}{c} \sqrt{\langle \Delta \lambda_{i}^{2}\rangle }=\sqrt{{\left(\Gamma^{-1}\right)}_{ii}}. \end{array}$$

Since there are nine parameters for a nonspinning MBHB, the Fisher information matrix is a 9 × 9 matrix. The sky location of the GW source is described by the sky coordinates (*θ*, *ϕ*) and the luminosity distance *d*_*L*_. The error in solid angle is given by

(25) $$\begin{array}{c} \Delta \Omega_{s}=2\pi \;|\;\sin\theta \;|\;\sqrt{\langle \Delta \theta^{2}\rangle \langle \Delta \phi^{2}\rangle -{\langle \Delta \theta \Delta \phi \rangle }^{2}}, \end{array}$$

where 〈Δ*θ*^2^〉, 〈Δ*ϕ*^2^〉, and 〈Δ*θ*Δ*ϕ*〉 are given by the inverse of the Fisher information matrix. In our analysis, we focus on the angular resolution and luminosity distance uncertainty. Although the Fisher information matrix gives a lower limit for parameter estimation, it is very helpful to estimate the localization capability for future experiments.

### 3.3. Mock Data Generation

We generate the mock data assuming a flat *Λ*CDM cosmology with *Ω*_*m*_ = 0.31, *Ω*_*Λ*_ = 0.69, and *H*_0_ = 67.74 km s^−1^ Mpc^−1^ [20]. Given a source redshift, we can easily calculate the luminosity distance and angular diameter distance to the source. Without loss of generality, we consider equal-mass black hole binaries with the total intrinsic masses of 10^5^ *M*_⊙_, 10^6^ *M*_⊙_, and 10^7^ *M*_⊙_, respectively. Note that the observed mass *M*_obs_ is related to the intrinsic mass *M*_int_ by the relation *M*_obs_ = (1 + *z*)*M*_int_. Since the intrinsic mass is degenerate with the redshift from GW measurements, the observed mass is used in our analysis.

LISA consists of a triangle of three spacecraft separated by 2.5 million kilometers while Taiji has three spacecraft with a separation distance of 3 million kilometers. Compared to LISA, Taiji is slightly more sensitive to low-frequency GWs. In our analysis, we adopt the noise power spectral density in the Michelson-style data channel for LISA obtained in [1] and for Taiji described in [2]. We ignore the foreground produced by millions of compact galactic binaries in our galaxy. The coalescence of MBHBs in general lasts for several days, months, or even years in the frequency band of LISA and Taiji. With the noise power spectral density, we calculate (22) choosing *f*_low_ = 0.4 mHz for the binary with a total intrinsic mass of 10^5^ *M*_⊙_, *f*_low_ = 0.1 mHz for the binary with a total intrinsic mass of 10^6^ *M*_⊙_, and *f*_low_ = 0.03 mHz for the binary with a total intrinsic mass of 10^7^*M*_⊙_.

The detector response functions and time delay between LISA and Taiji depend on the relative position of two detectors via *α* = 2*πf*_*m*_*t* + *κ*, which indicates that the angular resolution varies with the configuration angle *γ*. Given a redshift and total intrinsic mass of equal-mass black hole binaries, the GW signals are generated with random binary orientations and sky directions. To investigate the effect of the source position on the angular resolution, we simulate 10,000 random MBHB samples with the total intrinsic mass of 10^5^ *M*_⊙_ at a fixed redshift, assuming that *κ* = 0 for LISA and *κ* for Taiji is chosen in the range of [0, *π*]. Moreover, the sky location, binary inclination, polarization angle, and coalescence phase are randomly chosen in the range of *θ* ∈ [0, *π*], *ϕ* ∈ [0, 2*π*], *ι* ∈ [0, *π*], *ψ* ∈ [0, 2*π*], and *ϕ*_*c*_ ∈ [0, 2*π*], respectively. Without loss generality, the coalescence time *t*_*c*_ is set to be zero in our analysis.
